# N-Isopropylacrylamide-co-glycidylmethacrylate as a Thermoresponsive Substrate for Corneal Endothelial Cell Sheet Engineering

**DOI:** 10.1155/2014/450672

**Published:** 2014-06-09

**Authors:** Bernadette K. Madathil, Pallickaveedu RajanAsari Anil Kumar, Thrikkovil Variyath Kumary

**Affiliations:** Tissue Culture Laboratory, Biomedical Technology Wing, Sree Chitra Tirunal Institute for Medical Sciences and Technology, Poojappura, Trivandrum, Kerala 695012, India

## Abstract

Endothelial keratoplasty is a recent shift in the surgical treatment of corneal endothelial dystrophies, where the dysfunctional endothelium is replaced whilst retaining the unaffected corneal layers. To overcome the limitation of donor corneal shortage, alternative use of tissue engineered constructs is being researched. Tissue constructs with intact extracellular matrix are generated using stimuli responsive polymers. In this study we evaluated the feasibility of using the thermoresponsive poly(N-isopropylacrylamide-co-glycidylmethacrylate) polymer as a culture surface to harvest viable corneal endothelial cell sheets. Incubation below the lower critical solution temperature of the polymer allowed the detachment of the intact endothelial cell sheet. Phase contrast and scanning electron microscopy revealed the intact architecture, cobble stone morphology, and cell-to-cell contact in the retrieved cell sheet. Strong extracellular matrix deposition was also observed. The RT-PCR analysis confirmed functionally active endothelial cells in the cell sheet as evidenced by the positive expression of aquaporin 1, collagen IV, Na^+^-K^+^ ATPase, and FLK-1. Na^+^-K^+^ ATPase protein expression was also visualized by immunofluorescence staining. These results suggest that the in-house developed thermoresponsive culture dish is a suitable substrate for the generation of intact corneal endothelial cell sheet towards transplantation for endothelial keratoplasty.

## 1. Introduction

The cornea is the anterior transparent part of the eye and comprises five layers: the outer epithelium followed by the Bowman's membrane, stroma, Descemet's membrane, and finally the endothelium. The transparency of the cornea is maintained by regulating stromal hydration via the barrier and pump function of the single layered corneal endothelium. The corneal endothelium is a hexagonal nonreplicating monolayer of cells originating from the neural crest. Corneal endothelial dysfunction is the second leading cause of corneal blindness. Full thickness transplantation is the common mode of treatment of corneal endothelial dystrophies. Endothelial keratoplasty is a recent shift in surgical treatment wherein only the dysfunctional endothelium is replaced whilst retaining the unaffected layers [1]. This procedure has been reported to reduce the risks associated with penetrating keratoplasty mainly astigmatism and hemorrhage, while improving postoperative visual functions [2]. However this also requires the availability of donor corneas, the shortage of which still remains a limitation. To overcome this limitation, human corneal endothelial cells have been expanded* in vitro* onto carriers such as denuded Descemet's membrane, collagen matrix, amniotic membrane, human corneal stromal discs, gelatin hydrogel discs, and chitosan-based membranes [3]. However the use of carriers might facilitate contamination by pathogens and affect transparency of the cornea [4, 5].

Recent trends in tissue engineering of corneal endothelium are directed towards formation of transplantable cell sheets. Cell sheet technology enables the generation of carrier free tissue constructs via the use of stimuli-responsive surfaces. Poly(N-isopropylacrylamide) (pNIPAAm) grafted surfaces are the most commonly used thermoresponsive substrates for cell culture [6]. In an aqueous environment thermoresponsive surfaces exhibit reversible hydrophobic and hydrophilic properties above and below the lower critical solution temperature (LCST) facilitating the detachment of an intact cell sheet without the use of enzymatic treatments. The efficacy of pNIPAAm for generation of intact cell sheets for skin, cardiac, and periodontal reconstruction have been reported earlier [7, 8]. Copolymerization of pNIPAAm with glycidyl methacrylate allows modulation of LCST and also gives prospects of further modification by incorporation of biomolecules through the epoxy groups of the later [9]. The copolymer N-isopropylacrylamide-co-glycidylmethacrylate (NGMA) has been reported to be suitable to generate corneal epithelial cell sheets. This study further extends the possibility of NGMA in corneal tissue engineering by evaluating its potential in culturing corneal endothelial cells (CEC) and retrieval of functional corneal endothelial cell sheets.

## 2. Materials and Methods

### 2.1. Synthesis of Thermoresponsive Polymer NGMA

The NGMA was synthesized as previously described [9], from poly(N-isopropylacrylamide) (pNIPAAm) and glycidyl methacrylate (GMA) by free radical polymerization. Standard 35 mm diameter tissue culture polystyrene dishes were coated with the NGMA (4 g% in isopropanol) polymer, oven dried at 60°C overnight and sterilized using ethylene oxide.

### 2.2. Rabbit Corneal Endothelial Cell Sheet Engineering

Corneas from cadaveric New Zealand White rabbits (Sctb: NZW) were used in this study with prior approval of the Institute animal ethics committee. The rabbit corneas with sclera were collected in phosphate buffer containing (1000 IU/mL) penicillin and (1000 *μ*g/mL) streptomycin (Gibco, Invitrogen, India). The tissue was rinsed with buffer and sclera with limbus was removed. The endothelial side was treated with 0.25% trypsin (Gibco, Invitrogen, India) and explant cultures were initiated on the thermoresponsive substrate using Iscove's modified Dulbecco's medium (IMDM, Sigma, India) containing 10% FBS (Gibco), endothelial cell growth factor (Sigma, India), 100 IU/mL penicillin, and 100 *μ*g/mL streptomycin at 37°C in a 95% humidified atmosphere with 5% CO_2_. Explants were removed at the end of 48 hrs and the adhered endothelial cells were maintained up to four weeks in the incubator with media change on every third day. Corneal endothelial cells sheets with intact extracellular matrix were harvested by lowering the temperature below the LCST (28°C) of NGMA. Culture medium was replaced with 200 *μ*L serum-free IMDM and kept at 10°C for 1 min and then at 20–23°C for 5 min. The cells were monitored under phase contrast microscope (Leica DMI 6000, Germany) for detachment of cell sheet. Once the retrieval of cell sheet around the edges was notice, it was further removed either by swirling movement or mechanically with the help of a sterile polyethylene terephthalate sheet. The retrieved cell sheet was floated in PBS and the overlapped portions were carefully unfolded using pipette tips under a 3D stereomicroscope (Leica S8 APO, Germany).

### 2.3. Characterization of the Retrieved Corneal Endothelial Cell Sheet

#### 2.3.1. Scanning Electron Microscopy (SEM)

The morphology and architecture of the retrieved corneal endothelial cells sheet was evaluated by SEM. The cell sheet was washed thrice in phosphate buffer and fixed overnight in 3% glutaraldehyde. It was dehydrated in increasing concentrations of alcohol, critical point dried, and gold coated. The morphological detail of the corneal endothelial cell sheet was viewed under a scanning electron microscope (FEI Quanta 200, Germany).

#### 2.3.2. Viability of the Cell Sheet

The viability of the cell sheet was assessed by live-dead staining using fluorescein diacetate (FDA) and propidium iodide (PI). The cell sheet was treated with 5 *μ*g/mL of FDA (Sigma, India) in serum-free medium for 10 min followed by incubation with propidium iodide (0.05 *μ*g/mL) for one minute. The cell sheet was rinsed with phosphate buffered saline (PBS) and viewed under a fluorescence microscope (Leica DMI 6000, Germany).

#### 2.3.3. Immunofluorescence Staining

The cell sheet was fixed in 4% paraformaldehyde overnight. It was rinsed in PBS and permeabilized with 0.1% triton X100. Nonspecific binding sites were blocked with 1% bovine serum albumin (Sigma, India). The endothelial cell sheet was incubated with primary antibody mouse monoclonal to alpha-1 sodium potassium ATPase plasma membrane marker; 1 : 25 dilution, (Abcam, UK) overnight at 4°C and subsequently with FITC conjugated rabbit antimouse IgG; 1: 100 dilution (Sigma, India) for 1 h. Each incubation step was followed by washing with PBS. The cells were counterstained with Hoechst (Sigma, India) and viewed under a fluorescence microscope (Leica DMI 6000, Germany).

#### 2.3.4. Evaluation of Gene Expression by Reverse Transcriptase-Polymerase Chain Reaction (RT-PCR)

The cell sheet harvested was lysed and nucleic acids were extracted in 1 mL of TRI-solution (Merck-Bangalore Genei, India). The ribonucleic acid (RNA) was isolated by chloroform-isopropanol method and quantified. The complimentary deoxyribonucleic acid (cDNA) was synthesised using moloney murine leukemia virus reverse transcription polymerase chain reaction (M-MuLV RT-PCR, Merck-Bangalore Genei, India) kit. The target genes of the first strand cDNA was amplified by PCR (Genei Red dye PCR kit, Merck-Bangalore Genei) using their respective primers (Metabion GmbH, Germany) as described in Table 1. The target genes of collagen IV alpha-2 (Col-IV) and vascular endothelial growth factor receptor 2 (FLK1) were amplified for 37 cycles by touchdown PCR using the following parameters: 5 min at 94°C, 10 cycles of step-down PCR consisting of 1 min at 94°C, 50 s at 57°C then decrease by 0.5°C each cycle until 52°C; 1 min at 72°C, followed by 27 cycles of 1 min at 94°C, 50 s at 52°C, 1 min at 72°C, with a final extension of 5 min at 72°C. Aquaporin 1 and Na^+^-K^+^ ATPase target genes were amplified using the following parameters: 5 min at 94°C, 10 cycles of step-down PCR consisting of 1 min at 94°C, 50 s at 55°C then decrease by 0.5°C each cycle until 50°C; 1 min at 72°C, followed by 27 cycles of 1 min at 94°C, 50 s at 50°C, 1 min at 72°C, with a final extension of 5 min at 72°C. Beta-actin was used as control and the target gene was amplified for 30 cycles with annealing temperature of 52°C. All PCR processes were done in the thermocycler Eppendorf Mastercycler (EP Gradient S, Germany). The amplified PCR products were run on a 1.5% agarose gel containing 0.005% ethidium bromide and the bands were visualized using a Fluorescent Image Analyzer (Fuji FLA-5000).

## 3. Results

### 3.1. Corneal Endothelial Cell Sheet Retrieval

**Figure 1:**
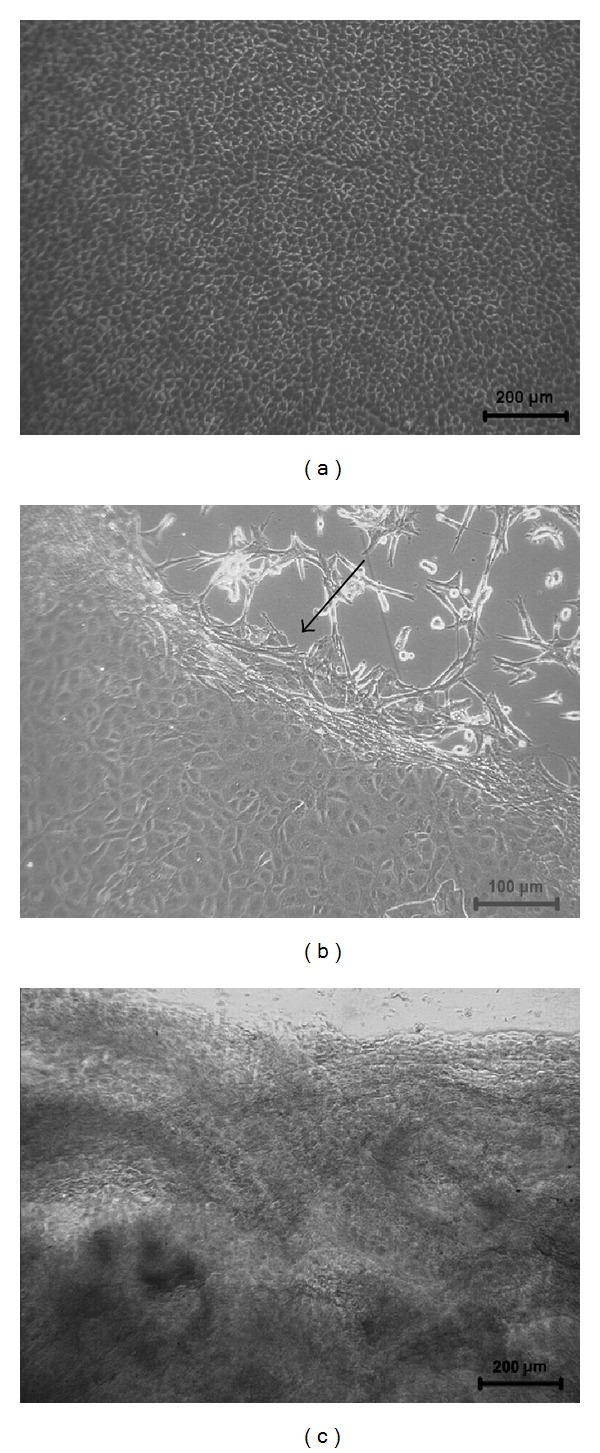
Phase contrast micrographs of the corneal endothelial cells before cell sheet retrieval (a); detachment of the cell sheet at 4°C after 15 min indicated with arrow (b); and retrieved cell sheet (c).

The explant culture of CEC established on NGMA exhibited characteristic cobble stone morphology. The cultures grew to confluence within seven days. Confluent cultures exhibited a single monolayer of closely apposed cells. The cells appeared to be more densely packed in the center of the culture (Figure 1). Following low temperature treatment at 4°C the cell sheet detaches from the periphery of the dish towards the center.

### 3.2. Morphology of the Corneal Endothelial Cell Sheet

**Figure 2:**
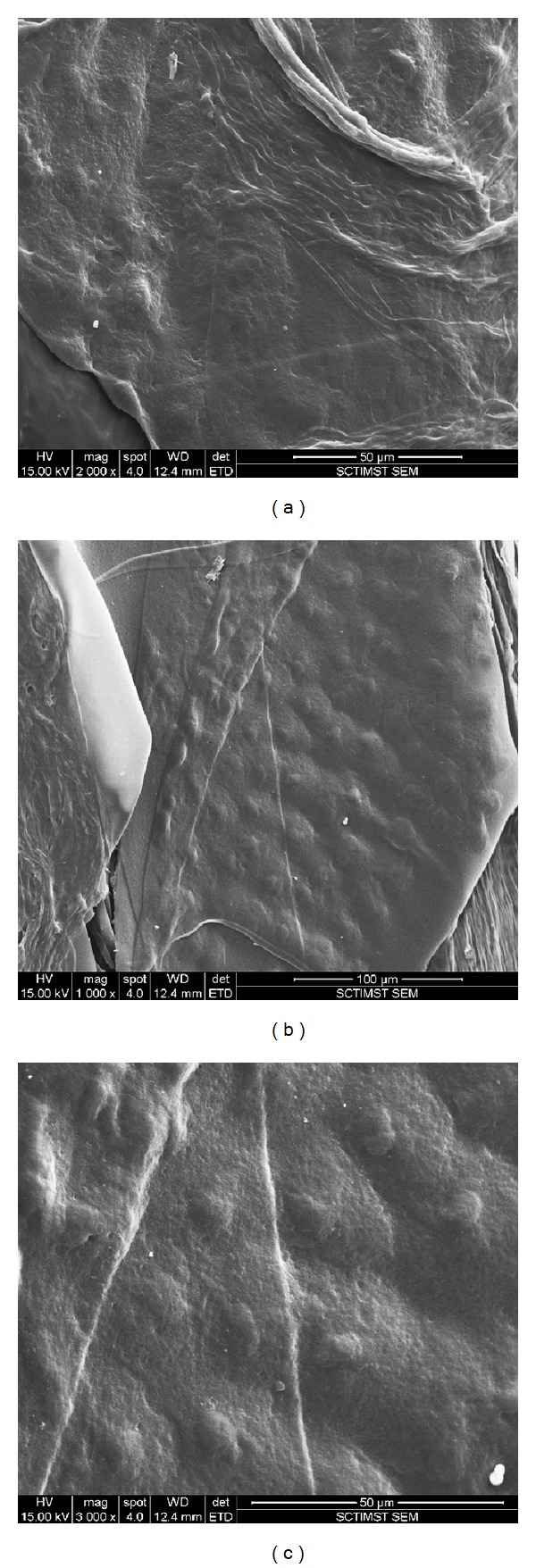
Scanning electron micrograph of the rabbit corneal endothelial cell sheet showing intact cellular morphology (a), extra cellular matrix deposition (b), and close apposition of cells (c).

SEM evaluation of the cell sheet revealed an intact architecture with good cellular morphology and cell-to-cell contact. The characteristic cobblestone morphology of the endothelial cells was observed along with profuse ECM deposition. This shows the NGMA coated tissue culture plates to be a feasible substrate for the nonenzymatic harvesting of intact cell sheets with the underlying ECM (Figure 2).

### 3.3. Viability of the Retrieved Cell Sheet

**Figure 3:**
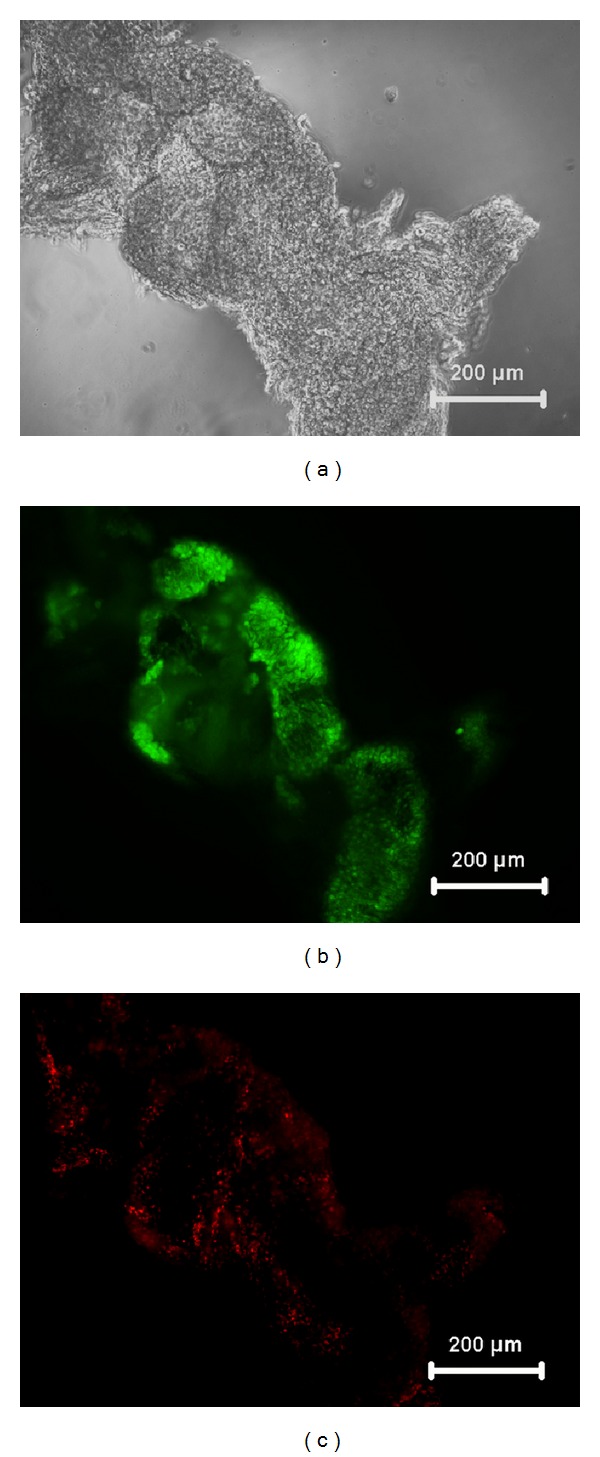
Corneal endothelial cell sheet immediately after retrieval by temperature variation (a), FDA-PI staining of cell sheet showing viable (green) cells (b), and very few nonviable cells (red) (c) under fluorescence microscope.

Viability of the retrieved cell sheet was confirmed by live-dead staining. Viable cells appeared green, while nonviable cells fluoresced red. Most of the cells in the retrieved cell sheet were viable indicating that the cell sheet retrieval procedure did not affect the viability of the cells (Figure 3).

### 3.4. Immunofluorescence

**Figure 4:**
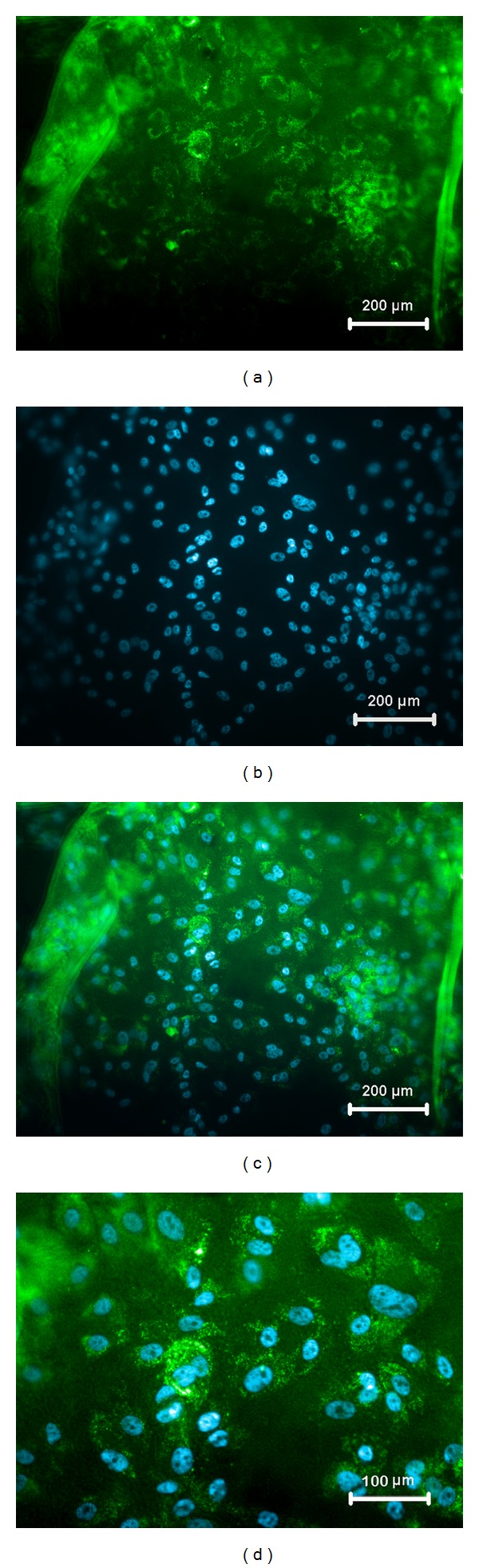
Immunofluorescence staining of the rabbit corneal endothelial cell sheet showed positive expression for the functional protein (a) Na^+^-K^+^ ATPase (green) counterstained with (b) Hoechst (blue). (c) shows merge of (a) and (b) and (d) is the 2x zoomed image of (c) showing characteristic staining.

Immunocytochemistry of the cell sheet revealed a positive staining for Na^+^-K^+^ ATPase which is a major functional protein involved in the pump function of the corneal endothelium (Figure 4). The expression was found to be mainly confined to the cellular borders.

### 3.5. Gene Expression in Corneal Endothelial Cell Sheet

**Figure 5:**
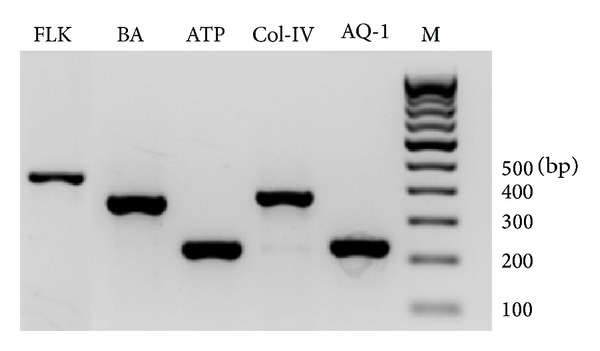
Expression of FLK-1, collagen IV (Col-IV), aquaporin-1 (AQ-1), Na^+^-K^+^ ATPase (ATP), and the housekeeping gene beta-actin (BA) in the rabbit corneal endothelial cell sheet as confirmed by RT-PCR (M-marker).

The gene expression of marker proteins FLK-1 and Col-IV and transmembrane proteins aquaporin-1 and Na^+^-K^+^ ATPase in the harvested cell sheet confirmed that the NGMA substrate does not inhibit the growth and function of the corneal endothelial cells (Figure 5). Furthermore, the low temperature treatment and cell sheet retrieval process also did not hinder the expression of these proteins.

## 4. Discussion

Tissue engineered corneal constructs are a potential therapeutic approach to treat diseases of the cornea. The recent techniques of endothelial keratoplasty enable the selective replacement of the diseased endothelium, whilst retaining the other corneal layers intact. Limitations in the availability of donor corneas have necessitated the need for alternatives like the use of bioengineered corneal endothelial constructs. Most approaches focus on the* in vitro* expansion of corneal endothelial cells onto synthetic or biological scaffolds like denuded Descemet's membrane, stromal disc, collagen matrix, gelatin hydrogel disc, chitosan-based membranes, and so forth [3]. However, transplanting such carrier to patient necessitates stringent quality control checks. Furthermore the biocompatibility and biological effects of degradation products of the carriers must be extensively evaluated.

The use of thermoresponsive substrates for* in vitro* cell cultures facilitates harvesting of cell sheet without the use of proteolytic enzymes like trypsin, dispase, and collagenase [10]. Hence the extracellular matrix (ECM), cell-cell contact are cell-ECM matrix interactions are maintained. We have earlier reported on the use of NGMA coated substrates for obtaining goat corneal epithelial cell sheets [9]. In the present study rabbit corneal endothelial cells were established on the in-house developed thermoresponsive substrate NGMA. The outgrowths from the explants adhered and grew to confluency without the addition of gelatin or other ECM coatings like fibronectin, chondroitin sulphate, and so forth. In most of the published data either minimum essential medium (MEM), Dulbecco's modified eagles medium (DMEM) or F99: Hams F:12 is used as the basal medium for* in vitro* culture of CEC [11]. Through this work we report for the first time the use of IMDM as the basal medium for establishing corneal endothelial cultures. Furthermore the CEC cultures were established directly onto the NGMA copolymer without the need for additional ECM coatings. Phase contrast microscopy revealed that CEC exhibits their characteristic cobblestone morphology with good cell-to-cell contact. Confluent cultures were achieved within a week.

Contact inhibition is an important mechanism by which corneal endothelial cells stop proliferation both* in vitro* and* in vivo*. These cells respond to mitogenic stimulation once cell-cell contact is broken either mechanically or enzymatically [12]. Although these cells are arrested in the G1 phase of the cell cycle they are actively maintained in this nonproliferative state [13]. These cells are metabolically active and secrete ECM components on their basal sides. In this study the rabbit corneal endothelial cell cultures were maintained* in vitro* for four weeks in order to allow ECM deposition to occur. This would aid in handling the cell sheet during the retrieval process. Intact corneal endothelial cell sheets were generated by subjecting the cultures to low temperature treatment at 4°C in presence of serum-free media. The principle of nonenzymatic harvest of cell sheets is that at temperatures below the LCST of NGMA, the culture surface changes from hydrophobic to hydrophilic. The polymer chains become hydrated and extended, enabling the lifting off of the corneal cell sheet. The use of serum-free media in this process prevents the rapid readhesion of the cell sheet to the underlying substratum. The retrieval procedure did not affect the viability of the cells as confirmed by FDA-PI staining. SEM evaluation of the retrieved cell sheet showed an intact cell-cell contact and strong ECM which was harvested along with the cell sheet. It has been earlier reported by Ide et al. that fibronectin and Col-IV are the main components of the Descemet's membrane secreted by the corneal endothelial cells* in vitro* [14]. Gene expression analysis of the harvested cell sheet shows the expression of Col-IV gene confirming the cellular activity of the cells sheet. Fan et al. have reported on the expression of FLK-1 (Vascular endothelial growth factor receptor-2) by corneal endothelial cells [15]. The gene expression of both marker proteins FLK-1 and Col-IV along with the phase contrast microscopy data confirmed that the* in vitro* cultures were of corneal endothelial cells.

The normal corneal thickness and transparency is maintained by the corneal endothelium via the action of membrane transport proteins like aquaporin 1, Na^+^-K^+^ ATPase, and voltage dependent anion channels (VDAC). Aquaporins (AQ) are water channels that facilitate the bidirectional osmotic water transport across the plasma membrane. Aquaporins 1, 3, and 5 are present in the cornea with AQ1 being mainly localized to the corneal endothelium and slight expression stromal keratocytes. Wen et al. has shown that AQ1 are localized to the apical and basolateral membrane domains of bovine corneal endothelial cells [16]. The Na^+^-K^+^ ATPase pump is a transmembrane enzyme that creates a sodium gradient by maintaining an internal low Na^+^ and high K^+^ concentration. This gradient developed facilitates to cotransport water and other ions across the plasma membrane. The Na^+^-K^+^ ATPase pump is reported to be localized to the basolateral membrane domain of the corneal endothelial cells [17]. Gene expression analysis of the retrieved cell sheet showed expression of both Aquaporin-1 and Na^+^-K^+^ ATPase. The characteristic membrane bound staining pattern of Na^+^-K^+^ ATPase was also observed by immunofluorescence. This establishes that the cells were able to withstand the retrieval process and intact barrier and pump functions were maintained in the harvested cell sheet.

## 5. Conclusion

In the present study rabbit corneal endothelial cells were cultured directly on the NGMA modified substrate without the need for any additional ECM coatings. The cultures could be maintained for prolonged periods and intact cell sheets could be harvested by simple variation of temperature. The cells sheets so obtained were found to have intact morphology and cell-to-cell contact. Gene expression analysis and immunocytochemistry confirmed the presence of functionally active cells. These results demonstrate the use of the in-house developed thermoresponsive polymer N-isopropylacrylamide-co-glycidylmethacrylate as a potentially good substrate for the generation of carrier free intact corneal endothelial cell sheets. However further studies on the functionality of the cell sheets by* in vitro* electrophysiological measurements and* in vivo* animal experiments are warranted. The presence of unreacted epoxy rings within the copolymer structure allows for the incorporation of biomolecules which could modulate cellular response on the thermoresponsive substrates.

## Figures and Tables

**Table 1:** Reverse transcription PCR primer sequence.

Sl. number	Name	Sequence (5′-3′)	Product length (bp)
1	Collagen IV A2 (Col-IV)	GTTCCAGGATGTGATGGACA (F) TCTCCAGTGTCACCTTTGTG (R)	371
2	Aquaporin 1 (AQ-1)	ACCTCGTCCCTGACCCTGAA (F) TGCCACAGCCAGTGTAGTCG (R)	215
3	Na^+^-K^+^ ATPase (ATP)	CTCTGTAACAGGGCGGTATT (F) ATTGGCGTTGAGGTTCTTAT (R)	219
4	FLK-1	ACGGAACATCCTCTTGTCGG (F) GCGCTCGCTTGTAACAGGTT (R)	410
5	Beta-actin (BA)	ATCGTGATGGACTCCGGCGA (F) AGGAAGGAGGGCTGGAAC (R)	350

## References

[1] Terry MA (2003). Deep lamellar endothelial keratoplasty (DLEK): pursuing the ideal goals of endothelial replacement. *Eye*.

[2] Mimura T, Yamagami S, Yokoo S (2012). Prospects for descemet stripping automated endothelial keratoplasty using cultured human corneal endothelial cells. *Journal of Transplantation Techniques & Research*.

[3] Mimura T, Yokoo S, Yamagami S (2012). Tissue Engineering of Corneal Endothelium. *Journal of Functional Biomaterials*.

[4] Dua HS, Azuara-Blanco A (1999). Amniotic membrane transplantation. *British Journal of Ophthalmology*.

[5] Hitani K, Yokoo S, Honda N, Usui T, Yamagani S, Amano S (2008). Transplantation of a sheet of human corneal endothelial cell in a rabbit model. *Molecular Vision*.

[6] Sumide T, Nishida K, Yamato M (2006). Functional human corneal endothelial cell sheets harvested from temperature-responsive culture surfaces. *The FASEB Journal*.

[7] Haraguchi Y, Shimizu T, Yamato M (2011). Regenerative therapies using cell sheet based tissue engineering for cardiac disease. *Cardiology Research and Practice*.

[8] Nagase K, Kobayashi J, Okano T (2009). Temperature-responsive intelligent interfaces for biomolecular separation and cell sheet engineering. *Journal of the Royal Society Interface*.

[9] Joseph N, Prasad T, Raj V, Anil Kumar PR, Sreenivasan K, Kumary TV (2010). A cytocompatible poly(*n*-isopropylacrylamide-*co*-glycidylmethacrylate) coated surface as new substrate for corneal tissue engineering. *Journal of Bioactive and Compatible Polymers*.

[10] Moran MT, Carroll WM, Gorelov A, Rochev Y (2007). Intact endothelial cell sheet harvesting from thermoresponsive surfaces coated with cell adhesion promoters. *Journal of the Royal Society Interface*.

[11] Parekh M, Graceffa V, Bertolin M (2013). Reconstruction and regeneration of the corneal endothelium: a review on the current methods and future aspects. *Journal of Cell Science & Therapy*.

[12] Senoo T, Obara Y, Joyce NC (2000). EDTA: a promoter of proliferation in human corneal endothelium. *Investigative Ophthalmology and Visual Science*.

[13] Joyce NC, Harris DL, Mello DM (2002). Mechanisms of mitotic inhibition in corneal endothelium: contact inhibition and TGF-*β*2. *Investigative Ophthalmology and Visual Science*.

[14] Ide T, Nishida K, Yamato M (2006). Structural characterization of bioengineered human corneal endothelial cell sheets fabricated on temperature-responsive culture dishes. *Biomaterials*.

[15] Fan T, Wang D, Zhao J, Wang J, Fu Y, Guo R (2009). Establishment and characterization of a novel untransfected corneal endothelial cell line from New Zealand white rabbits. *Molecular Vision*.

[16] Wen Q, Diecke FP, Iserovich P (2001). Immunocytochemical localization of aquaporin-1 bovine corneal endothelial cells and kertocytes. *Experimental Biology and Medicine*.

[17] Huang B, Blanco G, Mercer RW, Fleming T, Pepose JS (2003). Human corneal endothelial cell expression of Na^+^,K^+^-Adenosine triphosphatase isoforms. *Archives of Ophthalmology*.

